# Profile of the use of dermoscopy among dermatologists in Brazil (2018)^☆^^☆^

**DOI:** 10.1016/j.abd.2020.04.007

**Published:** 2020-07-15

**Authors:** Carlos Baptista Barcaui, Helio Amante Miot

**Affiliations:** a Department of Dermatology, Hospital Universitário Pedro Ernesto, Universidade do Estado do Rio de Janeiro, Rio de Janeiro, RJ, Brazil; b Department of Dermatology and Radiotherapy, Faculdade de Medicina, Universidade Estadual Paulista, Botucatu, SP, Brazil

**Keywords:** Brazil, Data collection, Dermatology, Dermoscopy

## Abstract

**Background:**

Dermoscopy increases the diagnostic accuracy in dermatology. The aspects related to training, usage profile, or perceptions of usefulness of dermoscopy among dermatologists in Brazil have not been described.

**Objectives:**

To evaluate the profile of the use of dermoscopy and the perception of the impact of the technique on clinical practice.

**Methods:**

The Brazilian Society of Dermatology invited all members to complete an online form with 20 items regarding demographic data, dermatological assistance, use of dermoscopy, and perceptions of the impact of the technique on clinical practice. The proportions between the categories were compared by analysis of residuals in contingency tables, and p-values < 0.01 were considered significant.

**Results:**

The answers from 815 associates (9.1% of those invited to participate) were assessed, 84% of whom were female, and 71% of whom were younger than 50 years of age. The use of dermoscopy was reported in the daily practice of 98% of dermatologists: 88% reported using it more than once a day. Polarized light dermoscopy was the most used method (83%) and pattern analysis was the most used algorithm (63%). The diagnosis and follow-up of melanocytic lesions was identified as the main use of the technique, while the benefit for the diagnosis of inflammatory lesions was acknowledged by less than half of the sample (42%).

**Study limitations:**

This was a non-randomized study.

**Conclusion:**

Dermoscopy is incorporated into the clinical practice of almost all Brazilian dermatologists, and it is recognized for increasing diagnostic certainty in different contexts, especially for pigmented lesions.

## Introduction

Dermoscopy is a non-invasive auxiliary method that increases the accuracy of the diagnosis of melanoma, as long as the dermatologist is adequately trained in the technique.^1^, ^2^, ^3^ Its use has been proven to reduce the number of unnecessary biopsies in benign lesions.^1^, ^4^, ^5^ For these reasons, dermoscopy is considered the standard method in the management of skin cancer and monitoring high-risk groups, and is included in current guidelines for clinical practice in several countries.^6^, ^7^, ^8^ In addition to its primary application in neoplasms, the number of indications for this technique in inflammatory diseases, infectious diseases, onychopathies, and alopecia is increasing.

The aspects related to training, usage profile, or perceptions of the impact of dermoscopy among dermatologists in Brazil have not been described. This study aimed to reveal the frequency and manner in which members of the Brazilian Society of Dermatology (Sociedade Brasileira de Dermatologia [SBD]) use and perceive the benefits of dermoscopy in their daily practice, and to describe the limiting factors for the use of this technique in Brazil.

## Methods

SBD invited all members to voluntarily answer an electronic survey (online form) containing 20 questions (Table 1), constructing a non-randomized sample (by adherence). The questions were prepared based on the study carried out in Europe by the International Dermoscopy Society, in addition to demographic and dermatological assistance data.^9^, ^10^

**Table 1:** Questionnaire submitted to members of the Brazilian Society of Dermatology (2018).

Questions	Answers:
1. What is your gender?	Female
	Male
2. How old are you?	
3. In which environment do you practice dermatology?	Private office
	Private hospital or clinic
	University hospital
	Public outpatient clinic
4. How long have you been practicing dermatology as a specialist?	
5. What is the number of patients you see per month?	
6. What is the mean number of skin cancer patients (all types) you see per month?	
7. Did you receive dermoscopy training as part of your residency or graduate degree in dermatology?	a) Yes
	b) No
8. In addition to your training during residency or graduate school, what type of training in dermoscopy did you undergo?	a) Dermoscopy course
	b) Online dermoscopy course
	c) Conferences/Congresses
	d) Books/Atlases
	e) Mentor/Tutor
	f) No training
9. Do you use dermoscopy in your daily practice?	a) Yes
	b) No
10. If you do not use dermoscopy, please indicate the reasons why not:	a) I do not consider it useful for my practice
	b) The equipment is very expensive
	c) The dermatoscope is not available in my office
	d) I do not have training in dermoscopy
	e) I am not confident enough in my skills for dermatoscopic diagnosis
	f) It takes too long
	g) It is not well reimbursed
	h) Others
11. How long have you been using dermoscopy?	a) < 2 years
	b) 2 − 5 years
	c) > 5 years
12. What type of dermatoscope do you use?	a) Non-polarized immersion contact (contact with skin, interface liquid, *e.g*., oil, alcohol)
	b) Polarized light dermatoscope
	c) Dermatoscope with digital camera
	d) Digital videodermoscopy (*e.g*., Fotofinder, Molemax, etc.)
13. In your daily practice, how often do you use dermoscopy?	a) Less than once/month
	b) 1 − 4 times/month
	c) More than once/week
	d) At least once/day
14. In your opinion, how useful is dermoscopy for the following?	a) Diagnosis of melanoma: Useful/Not very useful/Not useful
	b) Monitoring of melanocytic lesions: Useful/Not very useful/Not useful
	c) Diagnosis of pigmented tumors: Useful/Not very useful/Not useful
	d) Diagnosis of non-pigmented tumors: Useful/Not very useful/Not useful
	e) Diagnosis of inflammatory lesions: Useful/Not very useful/Not useful
	f. Follow-up of non-melanocytic skin lesions: Useful/Not very useful/Not useful
	g) Follow-up of non-melanocytic skin lesions: Useful/Not very useful/Not useful
15. When examining patients with the following skin problems, in what percentage of cases do you use dermoscopy:	a) Pigmented tumors: < 10% / 11%−30% / 31%−50% / 51%−70% / > 70% of cases
	b. Non-pigmented tumors: < 10% / 11%−30% / 31%−50% / 51%−70% / > 70% of cases
	ç. Inflammatory lesions: < 10% / 11%−30% / 31%−50% / 51%−70% / > 70% of cases
16. Which algorithm do you regularly use for the dermatoscopic diagnosis of pigmented lesions?	a) ABCD rule
	b) CASH
	c) Menzies algorithm
	d) Seven-point rule
	e) Pattern analysis
	f) I do not systematically use any particular algorithm
17. How confident are you in your dermoscopy skills for assessing the following types of lesions?	a) Pigmented tumors: Confident/Not very confident/Not confident
	b) Non-pigmented tumors: Confident/Not very confident/Not confident
	c) Inflammatory lesions: Confident/Not very confident/Not confident
18. In your opinion, the main advantages of using dermoscopy include: Strongly agree/Partially agree/Disagree/Indifferent	a) Diagnosis of early-stage melanoma
	b) Allows lesion follow-up
	c) Reduces the number of biopsies or unnecessary excisions
	d) Increases confidence in the clinical diagnosis
	e) Improves the way images are stored
	f) Reduces patient anxiety
	g) Improves documentation for legal purposes
	h) Increases remuneration
19. Do you think that the use of dermoscopy has increased the number of melanomas detected by you when compared with naked-eye examination?	a) Yes
	b) No
20. In your practice, how did the use of dermoscopy influence the number of excisions of benign lesions that you performed?	a) Decrease in the number of excisions of benign lesions
	b) Increase in the number of excisions of benign lesions
	c) No change in the number of excisions of benign lesions

The questionnaire was available online from June 27 to July 11, 2018. If the participant answered NO to question nine, the questionnaire was interrupted in question 10.

The answers were tabulated in MsExcel 2013, and assessed for duplications, anomalous values, and patterns of absence.^11^

The data of complete questionnaires were summarized as a percentage of responses (qualitative variables). Confidence intervals (95% CI) were calculated from 10,000 resamples (bootstrap).^12^ Quantitative variables were represented as mean and standard deviation (SD) or median and quartiles (p25 − p75), if normality was not assessed by the Kolmogorov-Smirnov test.^13^

The chi-squared test and the chi-squared test for trend were used to compare the proportions between the subgroups. Multinomial analyses were tested based on the analysis of residues in the contingency table. A p-value < 0.01 was considered significant.

## Results

The questionnaire was sent to all 8,884 SBD members and was answered in full by 9.1% (n = 815) volunteers. Table 2 presents the main demographic data of professional activity. The following were noteworthy: predominance of women, age less than 50 years, less than 20 years of professional activity, and greater representativeness of the states of the Southeast and South.

**Table 2:** Demographic and dermatological care data for the sample (n = 815).

Variable	Results
Sex – n (%)	
Female	681 (84)
Male	134 (16)
Age group – n (%)	
≤ 35 years	261 (32)
36 − 50 years	321 (39)
> 50 years	233 (29)
Time in dermatological practice – n (%)	
≤ 10 years	392 (48)
11 − 20 years	184 (23)
21 − 30 years	139 (17)
> 30 years	100 (12)
Geographical region – n (%)	
SE	501 (62)
S	136 (17)
NE	98 (12)
MW	47 (6)
N	23 (3)
Consultations per month – mean (SD)	224 (141)
Oncological consultations per month – median (p25−p75)	15 (8 − 30)
Type of practice – n (%)	
Private practice	720 (88)
Private hospital	274 (34)
Public outpatient clinic	243 (30)
University hospital	169 (21)

The main results related to the training and profile of use of dermoscopy are shown in Table 3. The use of dermoscopy in dermatological practice was reported by the vast majority of respondents (97.7%), with a high daily frequency. The reasons mentioned by those who do not use it were lack of confidence in the technique (26%, n = 5), not being well reimbursed by health plans (26%, n = 5), unavailability of equipment in the office (26%, n = 5), lack of training (21%, n = 4), considering it useless (16%, n = 3), considering the equipment expensive (16%, n = 3), taking too long (5%, n = 1), not being necessary to establish the diagnosis (5%, n = 1), considering that the magnifying glass is sufficient (5%, n = 1), and due to receiving patients already referred for excision (5%, n = 1).

**Table 3:** Data related to the training and profile of dermoscopy use (n = 815).

Variable	Results	95% CI^a^
Makes use dermoscopy – n (%)	796 (98)	97 − 99
Frequency of use – n (%)		
≥ Once per day	723 (88)	87 − 90
≥ Once per week	60 (7)	6 − 9
≥ Once per month	20 (3)	2 − 3
< Once per month	12 (2)	1 − 2
Dermoscopy device – n (%)		
Polarized light	677 (83)	81 − 85
Contact	320 (39)	36 − 42
Dermatoscope coupled to digital camera	162 (20)	17 − 22
Videodermoscopy	87 (11)	9 − 13
Dermoscopy training during medical residency – n (%)	489 (60)	57 − 63
Training in residency in relation to time in professional activity – n (%)		
≤ 10 years	355 (91)	88 − 93
11 − 20 years	90 (49)	42 − 55
21 − 30 years	29 (21)	14 − 26
> 30 years	15 (15)	8 − 21
Type of updating in dermoscopy – n (%)		
Book/Atlas	712 (87)	85 − 89
Congress/Conference	669 (82)	79 − 84
Classroom courses	662 (81)	78 − 83
Online courses	113 (14)	12 − 16
Mentor/Tutor	95 (12)	10 − 14
None	9 (1)	1 − 2
Diagnostic algorithm in pigmented lesions – n (%)		
Pattern analysis	515 (63)	60 − 66
No particular algorithm	143 (18)	15 − 20
ABCD	125 (15)	13 − 17
Menzies	18 (2)	1 − 3
Seven-point	14 (2)	1 − 3

a 95% confidence interval based on 10,000 resamples (bootstrap).

The frequency of training in dermoscopy during medical residency was higher for the respondents with shorter length of experience in the specialty (p < 0.01).

The formal means of continuing education in dermoscopy (books, classroom courses, and conferences) were the most used among those surveyed. The most widely used algorithm for diagnosing pigmented lesions was pattern analysis.

The perception of the usefulness of dermoscopy among the sampled dermatologists is shown in Table 4. Brazilian dermatologists value the usefulness of dermoscopy for the diagnosis of melanoma, monitoring of melanocytic lesions, and diagnosis of pigmented tumors; they underestimate its usefulness in the diagnosis of inflammatory lesions and in the follow-up of non-melanocytic lesions (p < 0.01).

**Table 4:** Perception of Brazilian dermatologists regarding the usefulness of dermoscopy (n = 815).

Variable – n (%)	Useful	Not very useful	Not useful
Diagnosis of melanoma	809 (99)^a^	5 (1)^b^	1 (-)^b^
Follow-up of melanocytic lesions	804 (99)^a^	10 (1)^b^	1 (-)^b^
Diagnosis of pigmented skin tumors	801 (99)^a^	12 (1)^b^	2 (-)^b^
Diagnosis of non-pigmented skin tumors	711 (87)	96 (12)^b^	8 (1)^b^
Follow-up of non-melanocytic lesions	532 (65)^b^	244 (30)^a^	39 (5)^a^
Diagnosis of inflammatory lesions	345 (42)^b^	407 (50)^a^	63 (8)^a^

Analysis of residuals in contingency table: ^a^*p* < 0.01 above expected; ^b^*p* < 0.01 below expected.

Expected values: useful (90%), not very useful (9%), not useful (1%).

a – above the expected, b – below the expected.

Table 5 presents the percentage of use of dermoscopy in tumors and inflammatory lesions. The frequency of use of dermoscopy in pigmented and non-pigmented tumors was higher than that of inflammatory dermatoses (p < 0.01).

**Table 5:** Percentage of use of dermoscopy in specific situations (n = 815).

Variable – n (%)	> 70%	31%−70%	11%−30%	< 10%
Pigmented tumors	583 (72)^a^	192 (24)^b^	20 (3)^b^	20 (3)^b^
Non-pigmented tumors	464 (57)^a^	252 (31)^b^	52 (6)^b^	47 (6)^b^
Inflammatory lesions	138 (17)^b^	321 (39)^a^	169 (21)^a^	187 (23)^a^

Analysis of residuals in contingency table: ^a^*p* < 0.01 above expected; ^b^*p* < 0.01 below expected.

Expected values: > 70% (48%); 31%−70% (31%), 11%−30% (10%), < 10% (10%).

Regarding the degree of confidence in the use of the technique for the diagnosis of inflammatory diseases and pigmented and non-pigmented tumors (Table 6), there was a lower degree of confidence in the use of dermoscopy in inflammatory lesions (p < 0.01).

**Table 6:** Degree of confidence in the use of dermoscopy for the diagnosis of pigmented tumors, non-pigmented tumors, and inflammatory diseases (n = 815).

Variable – n (%)	Confident	Somewhat confident	Not confident
Pigmented tumors	630 (77)^a^	172 (21)^b^	13 (2)^b^
Non-pigmented tumors	516 (63)^a^	261 (32)^b^	38 (5)^b^
Inflammatory lesions	209 (26)^b^	445 (55)^a^	161 (20)^a^

Analysis of residuals in contingency table: ^a^*p* < 0.01 above expected; ^b^*p* < 0.01 below expected.

Expected values: confident (55%), somewhat confident (36%), not confident (9%).

Table 7 presents the perception of associates regarding the advantages of dermoscopy in several applications. When compared with each other, an increase was observed in confidence in the clinical diagnosis, early diagnosis of initial melanoma, and lesion follow-up; in turn, only a partial perception of the documentation for legal purposes, storage of images, and reduction of the patients’ anxiety was observed. The increase in remuneration was not perceived by dermatologists as an advantage of using dermoscopy.

**Table 7:** Perception of the advantages of using dermoscopy according to the statements below (n = 815).

Variable – n (%)	I totally agree	I partially agree	I'm indifferent	I do not agree
Diagnosis early-stage melanoma	730 (90)^a^	81 (10)^b^	1 (−)^b^	3 (−)^b^
Allows lesion follow-up	758 (93)^a^	53 (7)^b^	1 (−)^b^	3 (−)^b^
Reduces biopsies or unnecessary excisions	641 (79)^a^	158 (19)	2 (−)^b^	14 (2)^b^
Increases confidence in clinical diagnosis	747 (92)^a^	66 (8)^b^	1 (−)^b^	1 (−)^b^
Improves how images are stored	573 (70)	173 (21)^a^	50 (6)	19 (2)^b^
Reduces patient anxiety	498 (61)^b^	248 (30)^a^	38 (5)	31 (4)^b^
Improves documentation for legal purposes	543 (67)	191 (23)^a^	53 (7)	28 (3)^b^
Increases remuneration	72 (9)^b^	215 (26)^a^	200 (25)^a^	328 (40)^a^

Analysis of residuals in contingency table: ^a^*p* < 0.01 above expected; ^b^*p* < 0.01 below expected.

Expected values: I totally agree (68%), I partially agree (19%), I’m indifferent (6%), I don't agree (7%).

In addition, 724 (88%) of respondents believed that dermoscopy promoted an increase in the number of diagnosed melanomas compared to naked-eye examination, and 660 (81%) reported that it reduced the excision of benign lesions.

## Discussion

This was a stimulated, non-randomized survey conducted by sending electronic communications to all SBD members. Despite the method, there was an adequate representation of the associates regarding sex, age group, and distribution among the geographical regions regions of the country. According to a 2017 survey, 78% of the members are female, the median age is 43 years, and the Southeastern and Southern regions of Brazil concentrate over 75% of the members.^14^

Regarding the environment in which the members practice dermatology, it is clear that many had more than one type of activity, with the vast majority (88%) working in private practices. Over 55% of the sample had been practicing dermatology for less than 15 years, reflecting a young society; 27% of the participants had been practicing the specialty for less than five years.

The total number of patients attended per month was quite heterogeneous among the interviewees, with a mean of 224 cases. It is noteworthy that half of the sample reported attending less than 15 cancer cases per month. From these data, skin cancer represents only 6.6% of the total dermatoses seen monthly by the dermatologist, which reflects the interest in areas of dermatology other than oncology. The customization of dermatological practice, such as cosmiatry, pediatric dermatology, or leprosy expertise, can represent the different impacts of dermoscopy on the individual reality of each professional.

Only 60% of the participants had some type of training in dermoscopy during their dermatology residency. From a historical perspective, dermoscopy is a relatively new method; despite being used since 1663 for the observation of nail capillaries, it only gained popularity in the late 1980s with the description of pattern analysis and the development of the portable manual dermatoscope.^15^, ^16^, ^17^ The first consensus on the terminology used in the English language was published in 1990; only recently there was a study published evaluating the reproducibility of these terms in the Portuguese language.^18^, ^19^ Nonetheless, the frequency of training in dermoscopy during residency was higher among participants with less time since graduation, showing the gradual incorporation of the technique into the training of new specialists.

The main reasons mentioned by those who do not use dermoscopy were lack of confidence in their skills and lack of training. The vast majority sought additional training at congresses, conferences, courses, and books. As it is a standard method for the management of skin cancer and is included in the current guidelines of clinical practice in several countries, it is essential to improve the formal teaching of this technique in the services accredited by the SBD. However, refresher courses offered at symposia and congresses play an important role in updating, especially for those who have been practicing dermatology for longer.

Among the reasons given by those who do not use dermoscopy, the fact that it is not well reimbursed by healthcare plans (26%) and the high price of equipment (16%) are worth mentioning. While those are irrelevant arguments from the scientific standpoint, they reflect the Brazilian reality and may suggest a greater role for the SBD as a class entity in championing its diagnostic procedures in state and private regulatory agencies.

The main type of dermatoscope used was the polarized light one (83%), which was expected given the portability and the hybrid use (polarized and non-polarized light) of current devices.

Regarding the usefulness of dermoscopy for the Brazilian dermatologist, the preference and the greater degree of confidence in the use in tumors, especially pigmented lesions, and the much less frequent use in inflammatory conditions were noteworthy. In fact, dermoscopy was popularized because it increased accuracy in the diagnosis of melanoma; however, the increasing use of the technique in trichology, onychology, and the diagnosis of infectious and inflammatory lesions should be promoted to improve the population's dermatological assistance.

Over 80% of respondents use pattern analysis or do not use algorithms in their daily practice, which probably reflects the degree of expertise and a selection bias in the sample studied. Simplified algorithms were developed so that non-experts could also diagnose melanoma, even at the expense of low specificity. For the dermatologist, pattern analysis is the method that best reflects the way in which the images are interpreted; moreover, it is the best method to teach dermoscopy for the diagnosis of melanoma by residents.^20^, ^21^

The limitations of the study are mainly the lack of randomization resulting from the spontaneous adherence of the invited dermatologists, which can impair the generalization of the data; however, this did not prevent consistent results from being unveiled.

Studies of patient care profiles and the use of technologies should be repeated periodically in order to subsidize dermatological medical education actions and to understand the assistance demands of the associates of dermatological societies.

## Conclusions

Dermoscopy has been incorporated into the clinical practice of almost all Brazilian dermatologists, especially those who are less than 50 years old and have practiced the profession for less than 20 years. Only 60% of Brazilian dermatologists received formal training in dermoscopy during their residency. The most widely used dermatoscope in Brazil is the polarized light device, while the most widely used diagnostic algorithm is pattern analysis. In the perception of the Brazilian dermatologist, dermoscopy is more beneficial for the diagnosis of neoplastic lesions, especially melanoma.

## Financial support

None declared.

## Authors’ contributions

Carlos Baptista Barcaui: Approval of the final version of the manuscript; conception and planning of the study; elaboration and writing of the manuscript; obtaining, analyzing, and interpreting the data; effective participation in research orientation; intellectual participation in propaedeutic and/or therapeutic conduct of studied cases; critical review of the literature; critical review of the manuscript.

Helio Amante Miot: Statistical analysis; approval of the final version of the manuscript; elaboration and writing of the manuscript; obtaining, analyzing, and interpreting the data; effective participation in research orientation; critical review of the literature; critical review of the manuscript.

## Conflicts of interest

None declared.
